# Stress and Microstructure Evolution in Mo Thin Films without or with Cover Layers during Thermal-Cycling

**DOI:** 10.3390/ma13183926

**Published:** 2020-09-04

**Authors:** Eunmi Park, Marietta Seifert, Gayatri K. Rane, Siegfried B. Menzel, Thomas Gemming, Kornelius Nielsch

**Affiliations:** 1 Leibniz IFW Dresden, Helmholtzstr. 20, 01069 Dresden, Germany; marietta.seifert@ifw-dresden.de (M.S.); rane.gk@gmail.com (G.K.R.); s.menzel@ifw-dresden.de (S.B.M.); t.gemming@ifw-dresden.de (T.G.); k.nielsch@ifw-dresden.de (K.N.); 2 Institute of Materials Science, TU Dresden, 01069 Dresden, Germany

**Keywords:** molybdenum thin films, high-temperature behavior, intrinsic stress

## Abstract

The intrinsic stress behavior and microstructure evolution of Molybdenum thin films were investigated to evaluate their applicability as a metallization in high temperature microelectronic devices. For this purpose, 100 nm thick Mo films were sputter-deposited without or with an AlN or SiO_2_ cover layer on thermally oxidized Si substrates. The samples were subjected to thermal cycling up to 900 °C in ultrahigh vacuum; meanwhile, the in-situ stress behavior was monitored by a laser based Multi-beam Optical Sensor (MOS) system. After preannealing at 900 °C for 24 h, the uncovered films showed a high residual stress at room temperature and a plastic behavior at high temperatures, while the covered Mo films showed an almost entirely elastic deformation during the thermal cycling between room temperature and 900 °C with hardly any plastic deformation, and a constant stress value during isothermal annealing without a notable creep. Furthermore, after thermal cycling, the Mo films without as well as with a cover layer showed low electrical resistivity (≤10 μΩ·cm).

## 1. Introduction

Accurate in-situ real-time temperature monitoring has become a key requirement to control and optimize processes in several industrial fields requiring high temperatures. The development of wireless and passive (i.e., operating without a battery) measurement systems has been strongly demanded to enable measurements at locations that cannot be connected by a wired sensor. Such parts are moving or rotating objects like turbine blades, which are exposed to an elevated temperature. Surface acoustic wave (SAW) devices have been widely used at medium temperatures as sensors for different applications, for instance, to monitor gas, strain, pressure, and temperature because of their advantages to be passive and robust and because they can be interrogated wirelessly [1,2]. SAW devices consist of patterned electrodes of a metallic thin film called interdigital transducers (IDTs), which are deposited on a piezoelectric substrate. One of the main issues for a SAW sensor to be used above 350 °C is the development of a suitable material for the IDTs that is able to withstand these high temperatures without degradation due to thermally induced processes like diffusion, agglomeration, oxidation, corrosion, and stress-induced defects. Aluminum-based IDTs, which are commonly used for conventional SAW devices, cannot be applied at higher temperatures due to softening and acoustomigration [3,4,5]. For the temperature range above 350 °C, platinum has attained interests due to its remarkable noble character and higher melting point T_m_ of 1768 °C, as compared to Al [6,7]. However, failures of the Pt thin film electrodes (thickness ≤ 100 nm) have also been reported at temperatures higher than 700 °C due to agglomeration effects [8,9]. Refractory metals with their high *T*_M_ could be good alternatives for the electrodes of the high temperature SAW devices, since a higher *T*_M_ leads to a reduction of degradation effects driven by diffusion and creep.

In our previous research, Ru-Al [10,11,12], Ti/Al [13], Mo/W [14], and oxide-dispersion-strengthened Mo-La_2_O_3_ [15] material systems were investigated regarding their suitability for applications in high temperature SAW devices, and the results showed their applicability as electrode materials for high temperatures.

Pure Mo thin films have attained interest in several technological areas [16,17] due to their high melting point (2623 °C) and the higher thermal conductivity of Mo (139 Wm^−1^K^−1^) [18] as compared to Pt (72 Wm^−1^K^−1^) [18]. Besides this, the electrical resistivity of Mo (5.5 μΩ·cm) [19] is lower than that of Pt (10.8 μΩ·cm) [19]. Thermal-stress-induced damages can be reduced because of the lower coefficient of thermal expansion (CTE) of Mo (4.8 × 10^−6^ at room temperature) [19], which is close to that of the applied substrate (3.3 × 10^−6^ at room temperature of Ca_3_TaGa_3_Si_2_O_14_ (CTGS) substrate [20]), as compared to the much higher CTE of Pt (8.8 × 10^−6^ at room temperature) [19]. In addition to the exceptional physical properties of Mo, mechanical properties, such as high strength and creep resistance, are notable advantages for their use as IDT materials for SAW temperature sensors [21]. However, there is a lack of knowledge of the thermomechanical behavior of Mo thin films at elevated temperatures. Therefore, we performed according measurements on isotropic Si (CTE 2.6 × 10^−6^ at room temperature [22]) substrates.

The mechanical behavior of thin films attached on a much thicker rigid substrate is different from that of their bulk counterparts in general. When the thin films are subjected to temperature changes, stresses are generated because of the mismatch in the CTE of the film and the substrate. Understanding and controlling the stresses in the films at the operating temperatures is challenging but important, to reduce the thermally induced drift and to improve the reliability, the long-term stability, and life-time of the high temperature sensors. Thermomechanical properties can be studied by measuring the stress behavior under thermal cycling. Face-centered cubic (FCC) metals like Cu [23,24,25], Al [26], and Ag [27] have been largely analyzed with this method, including the determination of the creep and relaxation behavior. On the other hand, the stress behavior of body-centered cubic (BCC) refractory metal thin films at elevated temperatures is unexplored yet.

In this paper, we report studies on the stress-temperature behavior, the thermal stability, and the electrical characteristics of 100 nm thin Mo films deposited on thermally oxidized Si substrates after annealing at up to 900 °C. In-situ stress measurement using a laser based multi-beam optical sensor (MOS) system was carried out under thermal cycling in ultrahigh vacuum (UHV) conditions up to 900 °C. As this is above a third of the melting temperature of Mo, the onset of plastic deformation effects can be expected.

## 2. Materials and Methods

Mo thin films with a thickness of 100 nm were prepared by DC magnetron sputtering in a high-vacuum (HV) chamber with a base pressure of 2.6 × 10^−6^ mbar at 400 °C of substrate temperature. A Mo target with 99.95% purity was used. The films were deposited onto 200 μm thick double side polished (100)-oriented single crystalline silicon substrates with 1000 nm of thermally grown silicon oxide on both sides, which acts as diffusion barrier between Si and Mo to prevent any unexpected reaction at elevated temperatures. Some of the Mo films were covered with 20 nm of AlN or SiO_2_. The AlN cover layer was deposited from a 100 mm AlN target by RF magnetron sputtering using a mixture of Ar and N_2_ gas with a ratio of 11:1. SiO_2_ was deposited from a SiO_2_ target by RF magnetron sputtering using a mixture of Ar and O_2_ gas with a ratio of 6:1. For the deposition of the SiO_2_ film, the substrate was heated to 180 °C. During the depositions, the substrate was rotated with 10 rpm to improve the homogeneity of the film thicknesses.

The samples (15 mm × 15 mm) were annealed for 24 h at 900 °C under UHV condition to stabilize their microstructure. Then, the films were subjected to a thermal cycling between room temperature and various temperatures (600, 700, 800, and 900 °C) to study their thermomechanical behavior. The heating and cooling rates were kept constant at 2 K/min, except for the cooling below 100 °C where the cooling rate was lower. All thermal treatments, including preannealing and thermal cycling, were done in UHV condition with a base pressure higher than 1.1 × 10^−9^ mbar to avoid oxidation. The residual stress and the thermal stress during the thermal cycling were measured by a kSA MOS in-situ stress monitoring system (k-space Associates, Inc. Dexter, MI, USA). The MOS system [28] consists of optical elements—etalons—which create an array of parallel laser beams with a 3 × 3 matrix from one laser source and are used to measure the film curvature in both the horizontal and vertical direction. The charge coupled device (CCD) camera measures the spacing between the reflected beams from the thin film surface, which is used to calculate the curvature and stress of the thin film. The average stress of the films was calculated as a mean value of the horizontal and vertical stress values. The in-situ curvature of the sample for each cycling was measured at 4 s intervals to monitor the stress changes. The residual stress of the as-deposited films was determined by measuring the change of the substrate curvature before and after deposition and calculated with Stoney’s equation [29]

(1) $$\sigma_{f}=\frac{E_{s}h_{s}^{2}}{6\left(1-v_{s}\right)h_{f}}\times \frac{1}{R},$$

where $\sigma_{f}$ is the stress in the film with the thickness of the film ($h_{f}$) and the substrate ($h_{s}$), Young’s modulus $E_{s}$ and the Poisson ratio $v_{s}$ of the substrate, and the measured radius of the curvature R.

The phase analysis of the samples was done before and after thermal cycling by X-ray diffraction (XRD, Philips x’Pert PW3040/00, Co-Kα, PANalytical, Almelo, The Netherlands) in Bragg–Brentano geometry. X-ray reflectivity (XRR) measurements were carried out on a Panalytical X’Pert MRD thin film device (Cu-Kα, PANalytical, Almelo, The Netherlands). The film roughness and density before and after thermal cycling were estimated by fitting the measured XRR profiles using the X’Pert Reflectivity program. The electrical resistance of the samples was determined by four-point probe measurements (van der Pauw method). The surface microstructural analysis was performed by scanning electron microscopy (SEM, Zeiss Ultra Plus, Carl Zeiss Microscopy GmbH, Oberkochen, Germany) and cross sections of the samples were prepared by the focused ion beam technique (FIB, Zeiss 1540 XB CrossBeam, Carl Zeiss Microscopy GmbH, Oberkochen, Germany) and imaged in the same device by SEM.

## 3. Results

**Figure 1:**
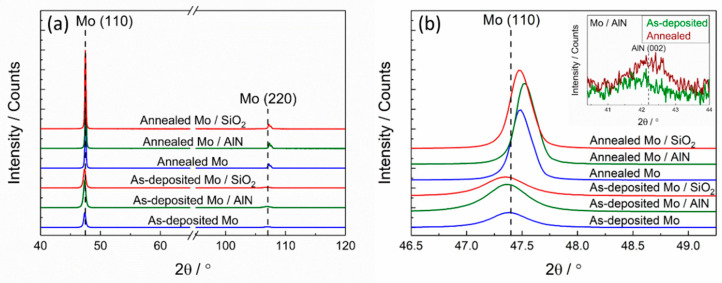
X-ray diffraction patterns of the Mo films with and without AlN or SiO_2_ cover layer in the as deposited state and after thermal treatment. (**a**) Full measurement regime; (**b**) zoom of the Mo (110) and AlN (002) peak (inset).

**Figure 2:**
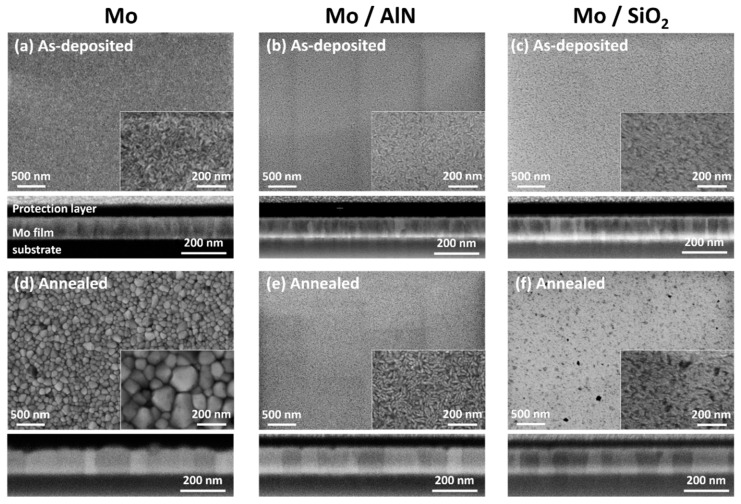
SEM (in-lens, 10 kV) micrographs and focused ion beam technique (FIB)-cut cross section views (in-lens, 3 kV) of (**a**) the as-deposited Mo film, (**b**) the as-deposited Mo film with AlN cover layer, (**c**) the as-deposited Mo film with SiO_2_ cover layer, (**d**) the annealed Mo film, (**e**) the annealed Mo film with AlN cover layer, and (**f**) the annealed Mo film with SiO_2_ cover layer. Cover layers (AlN and SiO_2_) are not visible in the FIB micrographs due to the low contrast between the cover layers and the FIB protection layer. The vertical stripes observed in (**b**,**e**) are caused by sample surface charging.

Figure 1a shows the X-ray diffraction patterns of the as-deposited and thermally cycled (up to maximum temperature of 900 °C) Mo films with and without AlN or SiO_2_ cover layer. The (110) and (220) diffraction peaks of the BCC structure of Mo are identified. Since the thermal treatments, including preannealing and thermal cycling, were performed in UHV condition, no oxidation was observed in the thermally cycled samples. After thermal cycling, in all samples, and uncovered and covered Mo films, a considerable increase of the intensity of the Mo peaks was observed due to the substantial grain growth during the preannealing, which is seen in the SEM images (Figure 2). Figure 1b shows the enlarged (110) Mo peaks. Broadened diffraction peaks with a low intensity are distinctly observed in the as-deposited Mo films with and with and without cover layer due to the small grain size and defects formed during sputtering. A strong increase in peak intensity is clearly visible as well as a shift of the peaks towards higher 2θ angles after thermal cycling. This shift of the Mo peak to higher angles as compared to the Mo powder diffraction peak position is attributed to the development of a tensile stress in thin films, which will be discussed below. If the Mo film was covered with a SiO_2_ layer, the Mo peaks were shifted to higher 2θ angles, similarly to that of the uncovered film. The shift of the (110) peak is stronger in case of the film with AlN cover layer, which is ascribed to a higher tensile stress in the AlN covered film. This is in good agreement with the results of the wafer curvature stress evaluation method, which will be discussed below. Besides this, the microstrain in the films was also evaluated by XRD single line broadening analysis [30], and the results are reported in Table 1. After thermal cycling, the microstrain values decreased in the films with and without a cover layer due to the relief of the defects. The inset in Figure 1b shows the AlN (002) peak of the Mo film covered with AlN layer. In the as-deposited state, the AlN (002) peak is hardly visible due to the very low intensity. After the thermal treatment, its intensity was slightly increased. This result can be interpreted by a structural rearrangement such as a crystallization of AlN during the thermal treatment from the amorphous-like structure in the as-deposited state.

Figure 2a–f show the SEM micrographs and FIB-cut cross section views of the as-deposited and annealed Mo films with and without cover layer. The as-deposited Mo film shows small rice-grain like crystallites with a predominant columnar structure (Figure 2a). After the thermal cycling, a significant grain growth leading to columnar grains spanning the entire film thickness with a largely increased but inhomogeneous in-plane grain size is visible in Figure 2d. The active surface diffusion during the grain growth resulted in an increase of the roughness. Figure 2b,c present the microstructure of the as-deposited Mo film with AlN or SiO_2_ cover layer, respectively, which show a similar surface morphology to the as-deposited state of the uncovered Mo film. A major difference between the annealed uncovered and covered films is that in the latter there was also stronger grain growth than in the as-deposited state, which was visible from the FIB-cut cross section images (Figure 2e,f), while the surface morphology hardly changed, which, in contrast, was the case in the uncovered films.

Despite the noticeable grain growth of the Mo, a smoother surface morphology than the uncovered film was maintained in all the covered films. This result indicates that the diffusion at the Mo/Cover interface is reduced compared with the free surface diffusion, so that in the covered films, the grain boundary diffusion is more important than other diffusion paths.

The increase in grain size and defect annihilation during annealing was accompanied by a reduction of resistivity (Table 1) independent of the presence of a cover layer.

**Figure 3:**
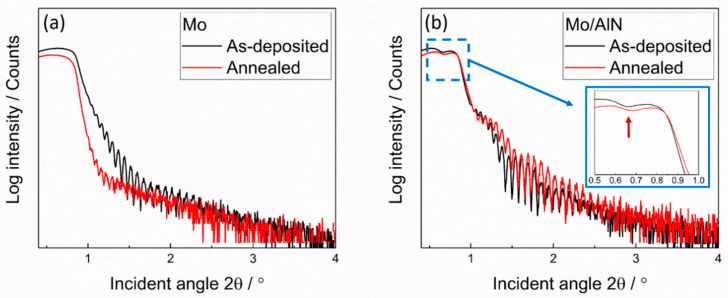
X-ray reflectivity (XRR) patterns for the as-deposited and annealed Mo films (**a**) without cover layer and (**b**) with AlN cover layer. The red arrow in inset image marks critical angle caused by AlN layer.

XRR measurements were additionally performed to obtain the film roughness and density (measurement data shown in Figure 3 and derived physical properties summarized in Table 1). The XRR measurements revealed a similar surface roughness for the as-deposited Mo film without cover layer (1.4 nm) and for the covered film (1.4 nm for film with AlN layer, 1.3 nm for the film with SiO_2_). The surface roughness of the covered Mo films was hardly changed during annealing, while that of the annealed uncovered Mo film was strongly increased (3.2 nm). Although a long-term preannealing of 24 h at 900 °C and several thermal cyclings were performed on the covered Mo films, the cover layers were not intermixed with the Mo film, i.e., both layers were maintained on the Mo film as separated layers. There was no noticeable change of the determined density of the Mo films within the measurement accuracy. In the XRR patterns of the film covered by AlN (Figure 3b), the first critical angle in the low angle regime caused by the AlN layer was shifted to a higher value after thermal treatment (marked with red arrow). This indicates that the density of the AlN cover layer was increased after thermal treatment (from 2.8 to 3.3 g/cm^3^, see Table 1), which is close to the density value of the bulk AlN of 3.26 g/cm^3^. Since the crystalline phase is denser than the amorphous one, this densification could be explained by the formation of an AlN crystalline phase [31], which was already indicated by the XRD measurements.

**Figure 4:**
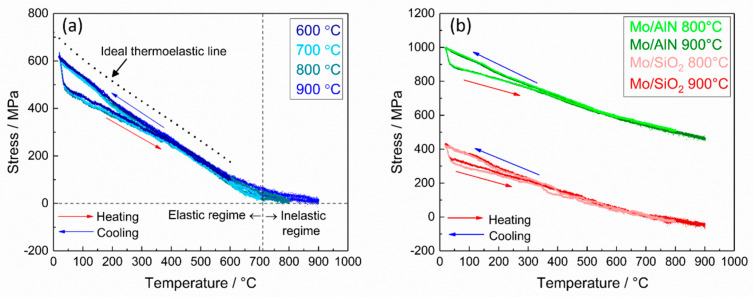
Stress as a function of temperature measured on the Mo films during the thermal cycles after preannealing: (**a**) uncovered Mo film and (**b**) covered Mo film with AlN or SiO_2_.

Figure 4a shows the result of the intrinsic stress measurements during the thermal cycling of the Mo films as a function of temperature after preannealing at 900 °C for 24 h (data of the preannealing not shown here), during which the grain growth of the Mo film took place and high tensile residual stress was developed (about 630 MPa). The plotted stress values were calculated from the measured sample curvature according to Stoney’s equation [29]. The uncovered Mo film was initially subjected to a thermal cycling between room temperature and 600 °C (Figure 4a). The drastic drop of the stress at the beginning of the heating was observed in all stress-temperature curves, and was caused by a temperature gradient due to the rapid increase in the heater current and a delayed response of the temperature control loop. However, this drop only occurs between room temperature and below 50 °C, which is a very low temperature with hardly any effect on the sample with such a high melting point. During the heating up to 600 °C, the stress-temperature curve shows an approximately linear behavior and the slope of the curve matches well with that of an ideal thermoelastic line, which is related to the coefficient of thermal expansion as follows:

(2) $$\mathrm{Slope}\;\mathrm{of}\;\mathrm{the}\;\mathrm{thermoelastic}\;\mathrm{line}=\;M_{f}\Delta \alpha ,$$

where *M_f_* is the biaxial modulus of the Mo film, which is 477 GPa, and Δ*α* is the difference of the thermal expansion coefficient between the Mo film and the Si substrate. The target temperature of the thermal cycling was increased to 700 °C, 800 °C, or 900 °C. Increasing the temperature up to 700 °C (~0.33 T_m_ of bulk Mo) results in a maximum radius of curvature of the sample, which means that the stress is reduced close to zero. At temperatures higher than 700 °C, the stress curves start to deviate from the thermoelastic line, which indicates the onset of an inelastic deformation. For the temperature range above 700 °C, the films maintain a low and almost constant stress level. Therefore, two regimes can be defined in the curves in Figure 4a: (1) a thermoelastic regime up to about 700 °C, in which the stress changes inversely proportional to the temperature due to the mismatch in the thermal expansion coefficient between the Mo film and the substrate, and (2) a plateau regime, in which the film undergoes an inelastic deformation at a nearly constant stress level. Analyses of FCC metal films such as aluminum or copper [27,32,33] at high temperatures above a third of the melting temperature revealed that their stress was approximately constant or only slightly changed as the temperature increased. In this plateau stress regime at high temperatures, diffusional creep was defined as the dominant inelastic deformation mechanism [34]. In the present work, we compare BCC Mo films with and without cover layers to study the influence of these cover layers on the diffusivity and with this on the thermal stress behavior.

Figure 4b shows the stress behavior during the thermal cycling up to 800 °C and 900 °C for the Mo films with AlN or SiO_2_ cover layer. As compared to the uncovered Mo film, the covered Mo film with an AlN layer shows a considerably higher residual tensile stress (about 1000 MPa) at room temperature after the thermal treatment (preannealing). The development of such high tensile stress during the preannealing can be explained by a material densification caused by crystallization [35], which was already concluded from the XRD and XRR results. The Mo film covered with a SiO_2_ layer shows a tensile residual stress (420 MPa) after preannealing as a consequence of grain growth, which is much lower than the other films. Therefore, it is concluded that the residual stress at room temperature after preannealing can be varied by changing the material of the cover layer.

The other distinctive discrepancy in the stress-temperature curve between covered and uncovered films is that the plateau regime, which is present at high temperatures above 700 °C in the uncovered film (regime 2 in Figure 4a), appears to be absent in both covered films, so that there is only the elastic deformation regime. This indicates that the cover layer reduces the inelastic deformation in the Mo film during thermal cycling by suppressing the diffusion at the surface of the Mo layer.

**Figure 5:**
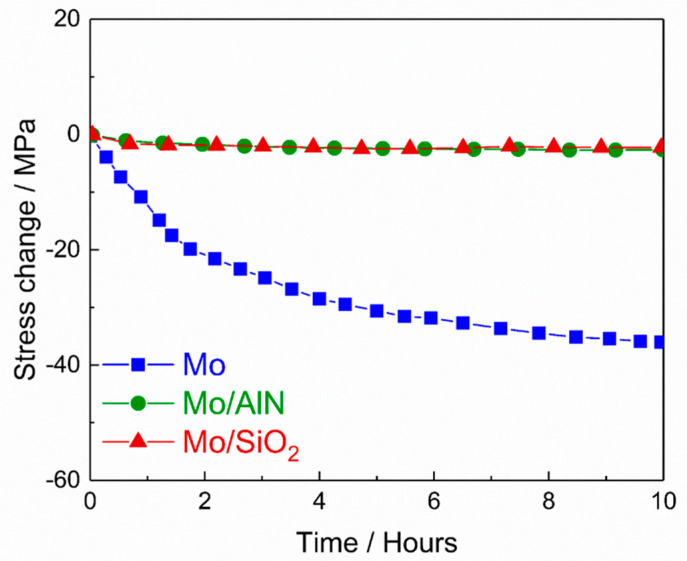
Isothermal measurements of the stress at 600 °C for the preannealed uncovered and covered Mo films (with AlN or SiO_2_ layer).

To study the effect of the cover layers on the intrinsic stress relaxation, Mo films with and without cover layer were isothermally held at a constant temperature (600 °C for 10 h) during the cooling phase. The results (Figure 5) are shown in the form of a change in stress as a function of time. Especially for the uncovered Mo film, a substantial stress relaxation took place. In the covered films, on the other hand, hardly any change of the stress was observed with time, even though the Mo film covered with AlN had a much higher stress energy than that of the uncovered Mo film. In general, stress can be relaxed by diffusion at grain boundaries and at the sample surface. However, since no significant stress relaxation was observed in covered films, the diffusion along grain boundaries alone is negligible for the relaxation in the cases presented here. A similar behavior was described, e.g., in [36].

## 4. Conclusions

In conclusion, the mechanical behavior of sputtered 100 nm thick Mo films with and without AlN or SiO_2_ cover layer was studied during thermal cycling up to 900 °C as well as during an isothermal annealing at 600 °C using a MOS in-situ stress monitoring system. In the uncovered Mo film, the stress-temperature behavior during thermal cycling can be divided into two regimes: the elastic behavior regime due to the mismatch in CTE between the Mo film and the Si substrate at low temperatures (T < 0.33 T_m_), and an inelastic deformation behavior regime by diffusional creep at high temperatures (T > 0.33 T_m_). The addition of the AlN cover layer on top of the Mo film has a significant effect, leading to a higher residual stress at room temperature after preannealing (900 °C for 24 h), and it reduces the plastic deformation at high temperatures. On the contrary, the film with SiO_2_ cover layer shows excellent stress behavior with a lower residual stress level at room temperature, and both covered films show a complete elastic behavior during thermal cycling. Isothermal annealing was carried out on the Mo films with and without cover layer. A significant relaxation was observed in the uncovered Mo film, while in the covered Mo films the stress maintains almost constant. The results indicate a mechanism of the stress relaxation in the Mo film, which is strongly dominated by surface diffusion. After thermal cycling, the Mo films with and without cover layer showed a grain growth accompanied by a significant reduction in resistivity as well as a stable microstructure without thermally induced damages. In summary, covered Mo films are suited for application as electrodes in SAW devices due to their low resistivity and the absence of relaxation processes.

## Figures and Tables

**Table 1:** Microstrain, density of the cover layer and Mo film, roughness, resistivity, and residual stress at room temperature for the as-deposited (AD) and thermally cycled (annealed) Mo films with and without AlN or SiO_2_ cover layer. (±0.1 g/cm^3^, ±0.06 nm, ±0.3 μΩ·cm and ±10 MPa of errors in density, roughness, resistivity, and stress measurements, respectively.).

		Mo		Mo/AlN		Mo/SiO_2_	
		AD	Annealed	AD	Annealed	AD	Annealed
Microstrain (%)		0.2	0.1	0.2	0.1	0.2	0.1
Density (g/cm^3^)	Cover layer	-	-	2.8	3.3	2.3	2.1
	Mo	10.2	10.0	10.2	10.1	10.2	10.2
Roughness (nm)		1.4	3.2	1.4	1.1	1.3	1.2
Resistivity (μΩ·cm)		13.2	8.1	13.2	8.5	13.9	9.0
Residual stress (MPa)		−250	630	−350	1000	−360	420
